# Identifying patterns of substance use and mental health concerns among adolescents in an outpatient mental health program using latent profile analysis

**DOI:** 10.1007/s00787-023-02188-7

**Published:** 2023-03-22

**Authors:** Jillian Halladay, Katholiki Georgiades, James MacKillop, Ellen Lipman, Paulo Pires, Laura Duncan

**Affiliations:** 1 The Matilda Centre for Research in Mental Health and Substance Use, University of Sydney, Australia Level 6, Jane Foss Russell Building, G02, Camperdown, NSW 2006 Australia; 2 Department of Psychiatry and Behavioral Neurosciences, McMaster University, 1280 Main Street West, Hamilton, ON L8S 4S4 Canada; 3 Offord Centre for Child Studies, McMaster University, Hamilton, Canada; 4 The Peter Boris Centre for Addictions Research, McMaster University/St. Joseph’s Healthcare Hamilton, 100 West 5Th St, Hamilton, ON L8N 3K7 Canada

**Keywords:** Adolescent, Cannabis, Alcohol, Tobacco, Mental health services

## Abstract

Though mental health and substance use concerns often co-occur, few studies have characterized patterns of co-occurrence among adolescents in clinical settings. The current investigation identifies and characterizes these patterns among adolescents presenting to an outpatient mental health service in Ontario, Canada. Data come from cross-sectional standardized patient intake assessments from 916 adolescents attending an outpatient mental health program (January 2019–March 2021). Latent profile analysis identified patterns of substance use (alcohol, cannabis, (e-) cigarettes) and emotional and behavioral disorder symptoms. Sociodemographic and clinical correlates of these patterns were examined using multinomial regression. Three profiles were identified including: 1) low substance use and lower frequency and/or severity (relative to other patients in the sample) emotional and behavioral disorder symptoms (26.2%), 2) low substance use with higher emotional and behavioral disorder symptoms (48.2%), and 3) high in both (25.6%). Profiles differed in sociodemographic and clinical indicators related to age, gender, trauma, harm to self, harm to others, and service use. Experiences of trauma, suicide attempts, and thoughts of hurting others increased the odds of adolescents being in the profile high in both substance use and symptoms compared to other profiles. These findings further document the high rates of substance use in adolescents in mental health treatment and the profiles generally map onto three out of four quadrants in the adapted four-quadrant model of concurrent disorders, indicating the importance of assessing and addressing substance use in these settings.

**Supplementary Information:**

The online version contains supplementary material available at 10.1007/s00787-023-02188-7.

## Introduction

Adolescents engaging with mental health services typically report higher levels of substance use than the general population [1, 2] and many adolescents who seek substance use treatment later on have previously received mental health services [3, 4]. The prevalence of substance use among grade 7–12 students across Ontario, Canada in 2019 was 14.8% for monthly heavy episodic drinking, 2% daily cannabis use, and 2% daily cigarette use [5]. In the same year, a study on adolescents admitted to an inpatient mental health unit in Ontario reported prevalence of these types and patterns of substance use at levels 2 to 9 times higher than the general student population [1]. Importantly, co-occurrence of substance use, even if not at the level of substance use disorder (SUD), and other mental health concerns is associated with more severe symptoms, greater complexity, poorer prognosis, and greater likelihood of suicidality [6, 7]. There have been national and international calls to action to improve early identification of substance use, and integrate efforts to address substance use and mental health concerns together among adolescents [8, 9]. Unfortunately, evidence and treatment gaps persist and there remains uncertainty regarding why, how, when, and for whom substance use and mental health concerns co-occur.

Common treatment for co-occurring substance use and mental health concerns includes the three following care models. *Sequential* treatment—where one concern is treated before the other, typically by different providers—is often criticized for ignoring the interconnectedness of problems. *Concurrent* treatment is where concerns are treated simultaneously by different providers which present challenges when treatment approaches differ or conflict, or communication between providers is absent. These barriers can be overcome with strong collaborative care. *Integrated* treatment is when care for both concerns is provided together by a single provider. Existing evidence suggests this is cost-effective, but it is incongruous with current training models and existing siloed healthcare and funding systems [3, 10, 11]. In caring for adolescents, it should be noted that substance use is often below the threshold of SUD [12] and may not require addiction-specific expertise to treat, making integrated approaches more feasible.

Cluster-based analyses can uncover non-linear patterns in associations between substance use and mental health concerns that may identify phenotypes of patients to inform care models [13, 14]. First, adolescents may use more than one substance. This is particularly common among hospitalized adolescents [1]. The types, frequency, and number of substances adolescents use may be related to both etiological and prognostic factors [13]. Second, substance use and mental health concerns overlap, though co-occurrence is not ubiquitous. Not all individuals with mental health concerns use substances and, at times, the presence of mental health concerns may decrease the likelihood of using substances [15]. Evidence suggests patterns of co-occurrence in adolescents map onto an adapted ‘four-quadrant model of concurrent disorders’ [13, 16], with low substance use and mental health concerns in quadrant 1, patterns of high in one but not the other in quadrants 2 and 3, and high in both in quadrant 4. However, existing studies use non-clinical samples, few include emotional disorder symptoms, and behavioral disorder symptoms are predominantly represented by single behavioral indicators. Examination of patterns in clinical samples is needed to uncover whether the four-quadrant model can be replicated, as focusing on individual substance use or mental health concerns separately—or assuming they consistently co-occur—may result in oversimplification and inaccurate inferences that misguide clinical decisions.

Once patterns of co-occurrence are identified and replicated, exploration of sociodemographic correlates (e.g., age, sex, gender, sexuality, race, and socioeconomic status) and clinical risk and complexity profiles are needed to guide contexts and targets for assessment and early intervention efforts. Substance use is typically higher among adolescents who are older [13], White or Multiracial [17], and experiencing socioeconomic disadvantage [18]. Female adolescents may be more likely to report co-occurring concerns [7], while transgender or gender diverse adolescents experience greater vulnerability to substance use and mental health concerns [19]. Both transgender and sexual minority adolescents experience significantly higher rates of suicidality [20]. Further, severe mental illnesses are often defined by the presence of psychotic symptoms, trauma-related disorders, and/or clinical indicators of acute risk including harm to self, harm to others, and need for hospitalization [21]. Characterizing co-occurrence patterns based on sociodemographic and clinical factors will help identify subpopulations requiring more and/or integrated support.

Our objectives were to characterize profiles of co-occurring substance use and mental health concerns in a clinical outpatient sample of adolescents. Identifying these patterns—or phenotypes—of adolescents presenting to mental health services may help quantify the need for and inform the development of integrated care models and/or collaborative care networks. More broadly, distinguishing and characterizing these different groups will help to inform future substance use prevention and treatment interventions.

## Methods

### Context and sample

Data come from self-report standardized assessments completed at intake to a specialized mental health outpatient program in a large urban city in Ontario, Canada. Presentations to this program are primarily driven by emotional concerns (e.g., anxiety, depression, and/or stress and trauma related disorders) and the service is generalized and does not provide specific SUD treatment. The sample for analysis includes 927 adolescents 12–17 years of age presenting between January 2019 and March 2021. This is a consecutively recruited sample of all adolescents attending the service during this period. The assessment is completed independently by the adolescents electronically (on a tablet in the waiting room prior to the pandemic and at home via email during the pandemic) before their first appointment. Their caregiver(s) are also provided with an intake questionnaire, completed independently and electronically (linked parent data ~ 848 for the 927 youth reports available). Completion rates for intake assessments are calculated for parent assessments of all children annually and were 96%, 74%, 77% in 2019, 2020, and 2021, respectively (youth specific response rates were unavailable). Use of these data was approved by Hamilton Integrated Research Ethics Board (#1355). Reporting follows the Strengthening the Reporting of Observational Studies in Epidemiology (STROBE) guidelines.

### Measures

#### Substance use

Adolescents were asked about past 6-month substance use with variables derived for frequency of alcohol, cannabis, and cigarettes, e-cigarettes, and tobacco [hereby referred to as (e-) cigarette/tobacco]. Frequency of alcohol use includes responses: never [0], not in the past 6 months [1], a few times [2], once a week [3], and 2 + times per week [4]. Frequency of cannabis use includes responses: never [0], not in the past 6 months [1], sometimes but not every day [2], at least once a day [3], more than once a day [4]. Frequency of (e-)cigarettes/tobacco was measured using a single combined item, and includes responses: never [0], not in the past 6 months [1], sometimes but not every day [2], at least once a day [3], and 10 or more times a day [4]. For other drug use, youth were asked*, “Have you ever used illegal or hard drugs such as cocaine, heroin or other types of illegal drugs?”* and responses were collapsed into a binary item reflecting past year use.

#### Emotional and behavioral disorder symptoms

Mental health concerns were assessed using the adolescent version of the Ontario Child Health Study Emotional Behavioural Scales (OCHS-EBS; [22]) that measure disorder symptoms in the past 6-months from never or not true (0) to often or very true (2) based on DSM-5 criteria. Subscale scores are summed where higher scores reflect more symptoms of disorder, including: emotional disorder symptoms (social phobia [SP], generalized anxiety [GAD], major depressive episode [MDE]) and behavioral disorder symptoms (attention deficit hyperactivity [ADHD], oppositional defiant [ODD], and conduct disorder [CD]). Confirmatory factor analyses confirmed the six-factor model was appropriate, and all subscales had adequate internal consistency (*a* > 0.73). See SM1 for more details.

#### Sociodemographic and clinical correlates

Sociodemographic and clinical characteristics came from adolescent and caregiver reports. Adolescent reported correlates included: age in years, gender [cis-gender girl/boy, transgender and gender diverse], LGBTQ status [1 = lesbian or gay, bisexual, transgender/transsexual/gender non-conforming, don't know, 0 = straight]; any lifetime physical or sexual abuse history, self-harm, suicidal ideation without attempt, suicide attempt(s), thoughts of hurting or killing others; and past 12-month mental health-related ED visit [0–1]. Caregiver-reported correlates included: race [1 = racial minority, 0 = White], household income [0–11; <  = 15 K to >  = 150 K], and concern about adolescent hearing or seeing things that are not there [psychosis; 0–1].

### Analysis

Profiles were identified through latent profile analysis (LPA) using Mplus (version 7) [23, 24]. Substance use [alcohol, cannabis, (e-)cigarette/tobacco] and emotional [SP, GAD, MDE] and behavioral [ADHD, ODD, CD] disorder symptom indicators were treated as continuous variables, where higher scores indicate more frequent substance use and a greater number and/or frequency of mental disorder symptoms in each emotional and behavioral domain. Models were estimated for 1-k profiles when the model no longer converged with up to 500 random starts or when Bayesian information criterion (BIC) began to increase. The best fitting model was selected by comparing solutions based on class enumeration and separation diagnostics, indicator specific class homogeneity and separation statistics, and theoretical clinical relevance of profiles. Class enumeration diagnostics included convergence, BIC and corrected Akaike’s information criterion (lower values and/or elbow on a line graph), approximate weight of evidence criterion (AWE), Lo-Mendell-Rubin adjusted likelihood ratio test (LMR-LRT), bootstrapped likelihood ratio test (BLRT), and relative improvement (RI) [24]. Models were also compared quantitatively and qualitatively based on clinical relevance of latent class separation, with quantitative class separation diagnostics including: posterior class probability (p), modal class assignment proportion (mcaP), average posterior probability (AvePP > 0.9), odds of correct classification (OCC > 5), and overall entropy (> 0.9) for the k-profile model [24]. Lastly, indicator specific class homogeneity and separation were also explored. Class homogeneity was examined by comparing within class indicator variance to the overall sample variance whereby ratios of > 0.9 indicate low homogeneity and < 0.6 indicate high homogeneity. Class indicator separation was examined using standardized mean differences (SMDs) for continuous indicators whereby SMDs > 2 indicated high separation and < 0.85 reflect low separation. Measurement invariance was confirmed pre-/post-COVID-19 and across gender. To note, emotional and behavioral disorder symptoms (EBS) scores are interpreted *relative* to the sample; ‘lower’ does not indicate low symptoms generally, but low relative to the full sample of adolescents.

All subsequent analyses use the most likely class membership. First, descriptive statistics were estimated across all profiles in SAS^®^ Enterprise Guide 7.1. Next, multinomial regression models were conducted to predict most likely profile membership in Mplus using full information maximum likelihood to account for missing data (missing data was minimal for self-reports, with highest due to missing caregiver reports [0–14.5%]). Models included: 1) a full sociodemographics model, and 2) a series of models examining clinical correlates (separately) controlling for sociodemographics. Odds ratios (ORs) of 1.44, 2.48, 4.27, and 8.82 are considered small, medium, large, and very large, respectively [25]; significant (*p *< 0.05) ORs >  = 2.48 are discussed in text.

## Results

Adolescents were 14.7 years of age on average, 53.4% were cis-gender girls, 14.2% were transgender or gender diverse, and 22.1% were of a racial minority. Full participant characteristics are shown in Table 1.

**Table 1:** Descriptive statistics reported as mean ± standard deviation or *n* (%)

	Outpatient (*n* = 927)	-SU/-EBS (*n* = 242)	-SU/ + EBS (*n* = 444)	+ SU/ + EBS (*n* = 236)
*Demographics*				
Age	14.7 ± 1.5	14.2 ± 1.6	14.6 ± 1.4	15.5 ± 1.2
Gender				
Cis-gender girl	495 (53.4%)	95 (39.3%)	252 (56.8%)	147 (62.3%)
Cis-gender boy	288 (31.1%)	129 (53.3%)	92 (20.7%)	67 (28.4%)
Transgender and gender diverse	132 (14.2%)	14 (5.8%)	96 (21.6%)	22 (9.3%)
LGBTQ status	393 (42.4%)	54 (22.3%)	237 (53.4%)	101 (42.8%)
Racial minority	205 (22.1%)	44 (18.2%)	113 (25.5%)	48 (20.3%)
Household income	5.7 ± 3.1	6.2 ± 3.3	5.5 ± 2.9	5.5 ± 3.1
*Profile indicators*				
Alcohol	0.8 ± 1.1	0.3 ± 0.7	0.4 ± 0.8	2.0 ± 1.0
Never	518 (55.9%)	187 (77.3%)	311 (70.0%)	19 (8.1%)
Not in the past	120 (12.9%)	25 (10.3%)	63 (14.2%)	32 (13.6%)
6 months	232 (25.0%)	25 (10.3%)	65 (14.6%)	142 (60.2%)
A few times	17 (1.8%)	2 (0.8%)	1 (0.2%)	15 (6.4%)
Once a week 2 + times a week	29 (3.1%)	0 (0.0%)	4 (0.9%)	28 (11.9%)
Cannabis	0.9 ± 1.3	0.2 ± 0.6	0.3 ± 0.6	2.6 ± 1.0
Never	572 (61.7%)	208 (86.0%)	358 (80.6%)	5 (2.1%)
Not in the past 6 months	73 (7.9%)	11 (4.5%)	47 (10.6%)	15 (6.4%)
Sometimes but not every day	153 (16.5%)	17 (7.0%)	34 (7.7%)	102 (43.2%)
At least once a day	55 (5.9%)	3 (1.2%)	0 (0.0%)	52 (22.0%)
More than once a day	62 (6.7%)	3 (1.2%)	0 (0.0%)	62 (26.3%)
(E-)Cigarette/Tobacco	0.9 ± 1.3	0.3 ± 0.7	0.3 ± 0.6	2.8 ± 1.1
Never	574 (61.9%)	197 (81.4%)	365 (82.2%)	11 (4.7%)
Not in the past 6 months	75 (8.1%)	19 (7.9%)	48 (10.8%)	8 (3.4%)
Sometimes but not every day	125 (13.5%)	19 (17.9%)	24 (5.4%)	82 (34.7%)
At least once a day	62 (6.7%)	2 (0.8%)	0 (0.0%)	60 (25.4%)
Ten or more times a day	80 (8.6%)	2 (0.8%)	3 (0.7%)	75 (31.8%)
Emotional disorder symptoms				
SP	6.7 ± 2.8	4.2 ± 2.6	8.0 ± 2.1	6.8 ± 2.5
GAD	8.2 ± 3.1	4.7 ± 2.5	9.8 ± 2.0	8.8 ± 2.7
MDE	10.5 ± 4.7	5.0 ± 3.3	12.5 ± 3.2	12.3 ± 3.6
Behavioral disorder symptoms				
ADHD	8.7 ± 3.7	5.7 ± 3.1	9.4 ± 3.3	10.4 ± 3.0
ODD	5.6 ± 2.9	3.7 ± 2.3	5.6 ± 2.7	7.4 ± 2.7
CD	3.2 ± 3.3	1.4 ± 1.9	2.6 ± 2.5	6.2 ± 3.9
*Clinical correlates*				
Other drug use	71 (7.7%)	2 (0.8%)	10 (2.3%)	59 (25.0%)
Trauma/Abuse	365 (39.4%)	44 (18.2%)	163 (36.7%)	158 (66.9%)
Self-harm	607 (65.5%)	77 (31.8%)	339 (76.4%)	190 (80.5%)
Suicidal ideation (no attempt)	232 (25.0%)	38 (15.7%)	138 (31.1%)	55 (23.3%)
Suicide attempt	365 (39.4%)	40 (16.5%)	55 (23.3%)	136 (57.6%)
Aggression	224 (24.2%)	25 (10.3%)	109 (24.5%)	90 (38.1%)
Psychosis	181 (19.5%)	36 (14.9%)	100 (22.5%)	45 (19.1%)
Prior ED visit	433 (46.7%)	75 (31.0%)	216 (48.6%)	142 (60.2%)

### Profile model selection

**Fig. 1:**
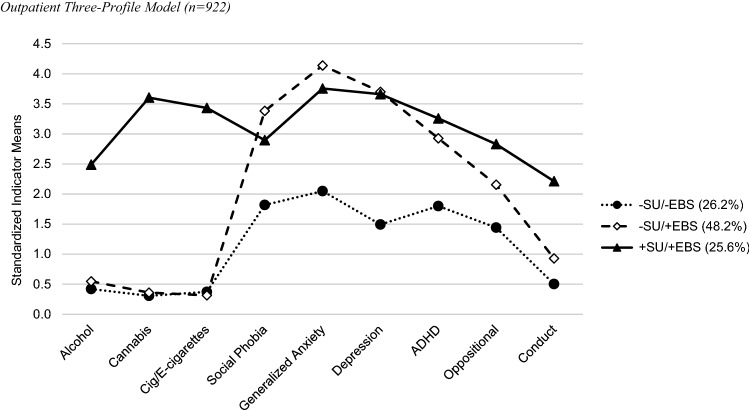
Outpatient three-profile model (n = 922)

The best fitting model was the three-profile model (Fig. 1) with an entropy of 0.89 and all posterior probabilities > 0.93 suggesting excellent fit with high classification certainty. The three profiles reflected: 1) low substance use, moderate emotional and ADHD, and low behavioral disorder symptoms profile (low substance use [-SU]/lower [*relative*] emotional and behavioral disorder symptoms [-EBS] representing 26.2% of the sample); 2) low substance use, high emotional and ADHD with moderate other behavioral disorder symptoms profile (-SU/ + EBS 48.2%); and 3) a high substance use and higher EBS profile (+ SU/ + EBS 25.6%). Fig. 1 reports standardized scores; please refer to Table 1 and Figure SM2.3 for raw scores. On average, adolescents in the + SU/ + EBS profile reported drinking occasionally in the past 6 months, using cannabis between sometimes and daily, and using (e-)cigarette/tobacco between sometimes and once a day. The symptoms in the + EBS profiles were above clinical thresholds, whereas adolescent in the -EBS profile reported subclinical symptoms on average.

### Sociodemographic and clinical correlates

See Table 2 for regression results (See SM3 for more comparisons). Twenty-five percent of adolescents in the + SU/ + EBS profile reported other drug use with < 3% in other profiles. Cis-gender girls had moderately higher odds of being in the -SU/ + EBS profile compared to the -SU/-EBS profile. Transgender and gender-diverse adolescents had the highest odds of being in the -SU/ + EBS profile, compared to both other profiles. Both profiles with higher mental health concerns, with and without substance use (-SU/ + EBS and + SU/ + EBS), had higher odds of endorsing lifetime self-harm, suicidal ideation, and attempts. Adolescents in the + SU/ + EBS had higher odds of past year ED visits, history of physical/sexual abuse, and thoughts of hurting/killing others in comparison to both other profiles, and higher odds compared to the -SU/ + EBS profile regarding suicide attempts.

**Table 2:** Regression results reported as odds ratio (95% confidence interval); *p* value

Reference = -SU/-EBS	+ SU/ + EBS	-SU/ + EBS
Sociodemographics		
Age	1.99 (1.71–2.32)**	1.22 (1.08–1.37)**
Cis-gender girl (ref = cis-boy)	2.35 (1.52–3.64)**	2.93 (2.00–4.30)**
Transgender and gender diverse (ref = cis-boy)	2.25 (0.97–5.25);0.06	5.48 (2.67–11.26)**
LGBTQ status	1.8 (1.12–2.89);0.015	2.16 (1.41–3.31)**
Racial minority	1.01 (0.60–1.70)	1.64 (1.08–2.51);0.021
Income	0.92 (0.86–0.99);0.023	0.95 (0.90–1.01)
Clinical correlates		
Abuse history	7.49 (4.75–11.83)**	1.99 (1.32–3.01);0.001
Self-harm	6.46 (4.05–10.29)**	4.48 (3.04–6.59)**
Suicidal ideation, no attempt (reference = no ideation or attempt)	4.12 (2.33–7.31)**	4.1 (2.58–6.51)**
Suicide attempt (reference = no ideation or attempt)	8.35 (4.99–13.98)**	4.19 (2.68 to 6.55)**
Thoughts of hurting or killing others	7.78 (4.42–13.71)**	3.03 (1.78–5.14)**
Psychosis	1.47 (0.87–2.48)	1.48 (0.92–2.36)
ED visit	2.78 (1.84–4.20)**	1.73 (1.21–2.47);0.003

*p *values of: < 0.001 reported as **; 0.06 < 0.001 exact value provided; > 0.06 not reported

## Discussion

This study identified distinct profiles of substance use and mental health concerns among a large sample of adolescents attending outpatient mental health services in Ontario, Canada. The identified profiles map onto three quadrants in the adapted four-quadrant model of concurrent disorders [13, 16]. The quadrant not identified was the high substance use but low symptom profile, likely due to the nature of the clinical program not being a designated concurrent disorders or substance use disorder program. Though the lower emotional and behavioral disorder symptom profile had average scores below clinical thresholds, these averages are still elevated in comparison to the general population [22] and may represent adolescents coming for early treatment, maintenance of previously treated symptoms, or who were presenting with unmeasured concerns. Adolescent reporting traumatic experiences, suicide attempts, and thoughts of hurting others had a higher odds of being in the profile high in both substance use and mental health concerns compared to other profiles, suggesting that co-occurrence may indicate greater clinical risk and complexity. Consistent with multidisciplinary clinical best practice guidelines [26–28] and recommendations from frontline staff [1], these results support the need to assess and address substance use among adolescents presenting to mental health services. This will require an integrated and/or shared care approach that may require adjustments or supplements to the current siloed models of clinical training, funding, and healthcare systems [3, 10, 11].

Trauma exposure was associated with 7.5 times the odds of being in profiles characterized by high levels of both substance use and mental health concerns compared to other profiles. This is consistent with research suggesting strong links between childhood trauma and substance use [29, 30] and research finding adolescent service users with trauma-related disorders report more substance use problems, even after adjusting for other mental disorders [2]. Hypotheses regarding the drivers behind comorbid trauma-related disorders and substance use problems include shared risk factors, self-medication of trauma-related symptoms with substances, and/or substance use contributing to higher risk contexts that place people at greater risk of trauma exposure [31, 32]. Findings suggest trauma and comorbid substance use may be important to address in adolescent mental health settings—there is an ongoing clinical trial in this area [32].

Though self-harm and suicidal ideation were similar across profiles with higher mental health concerns regardless of substance use status, suicide attempts were highest in the profile high in both substance use and mental health concerns (eight times the odds compared to the -SU/-EBS profile). This is consistent with literature suggesting that alcohol, cannabis, and/or cigarette smoking independently predict suicide attempts [6, 33, 34]. Further, the number of substances adolescents use also plays a role; in a large, representative, European study, every additional substance an adolescent reported using doubled their odds of reporting a suicide attempt [35]. Thus, substance use may provide a clinical clue for suicide risk, perhaps prompting thorough safety assessments and planning related to suicidality. Conversely, high suicidality may indicate a need to assess substance use in great depth and address it through integration with other treatment.

The profile with higher substance use had the highest co-occurring behavioral disorder symptoms and, accordingly, adolescents reporting thoughts of hurting or killing others had the greatest odds of being in the high in both substance use and mental health symptoms profile. This is consistent with strong, positive associations between substance use and externalizing symptoms in general population samples [30], substance use and externalizing disorders in clinical samples [2, 7], and evidence of bidirectional relationships between alcohol use and aggression in adolescence, whereby increases in one predicts increases in the other [36]. Aggression, substance use, and suicidality have all been characterized by difficulties with impulse control [37, 38], which could provide treatment directions.

Adolescents in the profile high in both substance use and mental disorder symptoms at intake to the outpatient program had a higher odds of reporting a past year ED visit for mental health concerns. Existing literature on substance-related predictors of representations to ED and readmissions is limited and conflicting. In recent reviews, SUDs were unexpectedly related to a lower likelihood of psychiatric readmissions among adolescents [39] with mixed findings related to re-presentations to ED among adolescents [40]. More work is needed to determine if and how co-occurring substance use is related to acute mental health service use, and these associations may differ based on the type of service. Given SUDs typically do not emerge until the mid-twenties [12], standardized instruments embedded into clinical care assessing substance use (not merely disorder) and mental health problems could help fill this gap [1, 41].

The only demographic characteristic uniquely, strongly related to the profile high in both substance use and emotional and behavioral symptoms was age, with older adolescents having a greater odds of being in this profile. This is consistent with incidence and prevalence of substance use increasing with age [5, 13, 42]. Further, adolescents who were cis-gender girls, transgender or gender diverse, or who endorsed LGBTQ status had higher odds of being in profiles characterized by more severe and frequent emotional and behavioral disorder symptoms (with or without substance use) [19, 20, 43]. However, these associations were stronger for the low substance use/high emotional and behavior disorder symptom profile than for the profile high in both. Similarly, youth mental health programs across Canada and other high-income countries similarly serve disproportionately high proportions of cisgender girls (55–64%), transgender and gender diverse youth (6–19%), and sexual minority youth (22–35%) [1, 44–47]. In a national survey of Canadian adolescents 15–17 years of age in 2019, 0.6% of adolescents reported being gender diverse and 21% reported being of a sexual minority [20]; thus, the prevalence of adolescents in this clinical sample reporting gender non-conformity was approximately 24 times higher and LGBTQ status was 2 times higher than the general population. This highlights the importance of considering minority stress models, practicing gender-affirming care, and creating welcoming, equitable, and accessible spaces for youth of all sexual and gender diversities in mental health care settings [48]. Overall, clinical severity and risk correlates yielded more pronounced associations with the substance use and mental health symptoms profiles than demographics in this sample.

## Limitations

First, the data are cross-sectional—causality and directionality cannot be inferred. Second, though adolescents were generally representative of adolescents that attend this service setting (predominantly presenting with emotional disorders), these data may not generalize to other mental health settings. The lack of association found for psychosis and profiles is likely a reflection of the clinical composition of the sample, although there is low incidence of psychosis in adolescence. Third, using measures from different informants may have suppressed some effect sizes, namely parent-reported psychosis. Fourth, we were unable to examine specific gender, sexual minority, or racial groups due to sample size, and the sexuality-related variable included a response related to gender, i.e., transgender (only 1.6% adolescents selected this response).

## Conclusion

This study found substance use and mental health symptom profiles among adolescents attending an outpatient mental health service that aligned with the adapted four-quadrant model of concurrent disorders [13]. Adolescents presenting to these services with elevated substance use and mental health concerns disproportionately endorsed a history of trauma, suicide attempts, and aggression in comparison to other profiles. This work suggests substance use may be an indicator of greater clinical risk, making integrated and collaborative care models important to consider in adolescent mental health settings.

## Supplementary Information

Below is the link to the electronic supplementary material.

Supplementary file1 (DOCX 242 KB)

## Data Availability

The data used for this study are not publicly available due to its sensitive clinical nature. Data are available from the corresponding author upon reasonable request and will be subject to further ethics approval.

## Code availability

Available upon request from the corresponding author.
