# Supplementary material for: Identifying patterns of substance use and mental health concerns among adolescents in an outpatient mental health program using latent profile analysis
Source: Eur Child Adolesc Psychiatry. 2023 Mar 22;33(3):739–47. doi: 10.1007/s00787-023-02188-7 (PMC10031175; doi:10.1007/s00787-023-02188-7)
Supplement: Supplementary file 1 — Supplementary file1 (DOCX 242 KB) [file 787_2023_2188_MOESM1_ESM.docx]

**Supplementary Materials**

**SM1. Confirmatory Factor Analysis of Mental Health Symptom Scales**

Table S1.1 Mental health CFA model fit indices. All models used mean and variance adjusted diagonally weighted least squares (WLSMV) estimation with delta parameterization.

| Model | X^2^ (df) | CFI | RMSEA (90% CI) | WRMR |
| --- | --- | --- | --- | --- |
| 2 Factor (INT and EXT) | 6055.2 | 0.83 | 0.077 (0.075, 0.079) | 2.94 |
| 6 factor (SP, GAD, MDE, ADHD, ODD, CD) | **3654.0** | **0.91** | **0.056 (0.054, 0.058)** | **2.07** |
| 6 Factor (<0.6 loadings removed) | 2940.7 | 0.92 | 0.058 (0.055, 0.060) | 2.04 |

Table S1.2 Average Variance Extracted compared to Interfactor Correlations

| Domain | AVE(√AVE) | Interfactor Correlations | | | | | |
| --- | --- | --- | --- | --- | --- | --- | --- |
|  |  | SP | GAD | MDD | ADHD | ODD | CD |
| SP | 0.82 | 1 |  | | | | |
| GAD | 0.80 | 0.72 | 1 |  | | | |
| MDD | 0.77 | 0.57 | 0.75 | 1 |  | | |
| ADHD | 0.69 | 0.33 | 0.46 | 0.50 | 1 |  | |
| ODD | 0.70 | 0.17 | 0.28 | 0.42 | 0.68 | 1 |  |
| CD | 0.70 | 0.01 | 0.10 | 0.33 | 0.59 | **0.89** | 1 |

Table S1.3. Standardized item loadings and internal consistency in separate factor models

| Variable name | Question | Standardized loading |
| --- | --- | --- |
| **Social Phobia (SP)** | | |
| Cronbach alpha | | 0.85 |
| OCHS1 | I am afraid of doing things in front of others | 0.82 |
| OCHS6 | I avoid social situations | 0.75 |
| OCHS16 | I don’t like to be with people I don’t know well | 0.74 |
| OCHS22 | I get anxious about meeting new people | 0.90 |
| OCHS32 | I feel nervous with people I don’t know well | 0.89 |
| **Generalized Anxiety Disorder (GAD)** | | |
| Cronbach alpha | | 0.84 |
| OCHS3 | I am anxious or on edge | 0.84 |
| OCHS21 | I find it hard to stop worrying | 0.84 |
| OCHS35 | I am nervous or tense | 0.91 |
| OCHS44 | I am too fearful or anxious | 0.91 |
| OCHS49 | When anxious, my mind goes blank | 0.57 |
| OCHS50 | I worry about doing better at things | 0.66 |
| **Major Depressive Episode (MDE)** | | |
| Cronbach alpha | | 0.87 |
| OCHS10 | I have had a change in appetite | 0.56 |
| OCHS13 | I deliberately try to hurt or kill myself | 0.78 |
| OCHS19 | I feel worthless or inferior | 0.85 |
| OCHS25 | I get no pleasure from usual activities | 0.71 |
| OCHS30 | I have trouble enjoying myself | 0.82 |
| OCHS37 | I feel overtired or lack energy | 0.77 |
| OCHS43 | I think about killing myself | 0.82 |
| OCHS45 | I have trouble sleeping | 0.66 |
| OCHS47 | I am unhappy, sad or depressed | 0.89 |
| **ADHD** | | |
| Cronbach alpha | | 0.81 |
| OCHS8 | I have trouble concentrating or paying attention | 0.77 |
| OCHS9 | I can’t stay seated when required to do so | 0.61 |
| OCHS15 | I am easily distracted, have difficulty sticking to any activity | 0.74 |
| OCHS18 | I fail to finish things I start | 0.74 |
| OCHS20 | I fidget | 0.63 |
| OCHS28 | I have difficulty awaiting my turn in games or groups | 0.49 |
| OCHS31 | I act without stopping to think | 0.70 |
| OCHS34 | I make careless mistakes | 0.80 |
| **Oppositional Defiant Disorder (ODD)** | | |
| Cronbach alpha | | 0.78 |
| OCHS2 | I am angry and resentful | 0.78 |
| OCHS4 | I argue a lot with adults | 0.68 |
| OCHS7 | I blame others for my own mistakes | 0.53 |
| OCHS17 | I am easily annoyed by others | 0.69 |
| OCHS23 | I get back at people | 0.69 |
| OCHS33 | I lose my temper | 0.82 |
| **Conduct Disorder (CD)** | | |
| Cronbach alpha | | 0.79 |
| OCHS12 | I am mean to others | 0.78 |
| OCHS14 | I destroy things belonging to others | 0.69 |
| OCHS24 | I get in many fights | 0.83 |
| OCHS26 | I have been physically cruel to others | 0.72 |
| OCHS27 | I have broken into someone else’s house, building or car | 0.73 |
| OCHS38 | I run away from home | 0.67 |
| OCHS40 | I set fires | 0.63 |
| OCHS41 | I stay out at night despite being told not to | 0.61 |
| OCHS42 | I steal things from places other than home | 0.72 |
| OCHS46 | I cut classes or skip school | 0.57 |
| OCHS48 | I use weapons when fighting | 0.68 |

Table S1.4. Correlations using summative scores that were used as indicators in subsequent LPA

|  | ALC | CAN | (E-)CIG | SP | GAD | MDE | ADHD | ODD | CD |
| --- | --- | --- | --- | --- | --- | --- | --- | --- | --- |
| ALC | 1 |  |  |  |  |  |  |  |  |
| CAN | 0.65 | 1 |  |  |  |  |  |  |  |
| CIG | 0.62 | 0.70 | 1 |  |  |  |  |  |  |
| SP | -0.03 | 0.01 | -0.03 | 1 |  |  |  |  |  |
| GAD | 0.10 | 0.09 | 0.08 | 0.60 | 1 |  |  |  |  |
| MDE | 0.18 | 0.21 | 0.19 | 0.49 | 0.66 | 1 |  |  |  |
| ADHD | 0.17 | 0.21 | 0.25 | 0.26 | 0.38 | 0.44 | 1 |  |  |
| ODD | 0.25 | 0.31 | 0.32 | 0.13 | 0.22 | 0.34 | 0.54 | 1 |  |
| CD | 0.40 | 0.47 | 0.48 | 0.02 | 0.10 | 0.29 | 0.44 | 0.65 | 1 |

**SM2. LPA Model Enumeration and Evaluation**

**Expanded Methods:** Substance use and mental health profiles were identified through Latent Profile Analysis (LPA) using Mplus (version 7). All substance use and mental health indicators were treated as continuousvariables. Models were estimated for 1 profile upto k profiles when the model no longer converged with up to 500 random starts or when Bayesian Information Criterion (BIC) began to increase (Collins & Lanza, 2009; Geiser, 2012; Masyn, 2013). The following class enumeration diagnostics were compared across models: convergence, BIC and Corrected Akaike’s Information Criterion (CAIC; both assessing for smaller scores and elbow on a line graph of estimates), Approximate Weight of Evidence Criterion (AWE), Lo-Mendell-Rubin adjusted likelihood ratio test (LMR-LRT), bootstrapped likelihood ratio test (BLRT), and Relative Improvement (RI) (Masyn, 2013). Models were also compared quantitatively and qualitatively based on clinical relevance of latent class separation, with quantitative class separation diagnostics including: posterior class probability (p), model class assignment proportion (mcaP), average posterior probability (AvePP >0.9), odds of correct classification (OCC>5), and overall entropy (>0.9) for the k-profile model (Masyn, 2013). Lastly, indicator specific class homogeneity and separation were also explored. Class homogeneity was examined by comparing within class indicator variance to the overall sample variance whereby ratios of >0.9 indicate low homogeneity and <0.6 indicate high homogeneity. Class indicator separation as examined using standardized mean differences (SMDs) for continuous indicators whereby SMDs >2 indicated high separation and <0.85 reflect low separation.

Measurement invariance comparing pre-COVID-19 (January 2019 to February 2020) and COVID-19 (March 2020 to March 2021) times was then examined by: 1) stratifying the sample into pre-COVID-19 and COVID-19 and re-estimating best fitting models, and 2) using multi-group functioning where groups were i) constrained to have equal parameter estimates versus ii) freed parameter estimates (Collins & Lanza, 2009; Masyn, 2013). Similar approaches were used to assess gender invariance. Models were compared based on BIC and CAIC, AWE. Models were also compared quantitatively and qualitatively based on clinical relevance of latent class separation. Of note, in the context of measurement invariance, the principle of parsimony refers to the full multiple group solution (not the number of classes within groups).

Table S2.1 Model fit

| k-classes | LL | Npar | BIC | CAIC | AWE | LMR-LRT p-value | BLRT p-value | Relative Improvement |
| --- | --- | --- | --- | --- | --- | --- | --- | --- |
| **Full sample (n=922)** | | | | | | | |  |
| 1 | -18952.1 | 18 | 38027.09 | 37975.57 | 37984.57 |  |  |  |
| 2 | -17903.5 | 28 | 35998.12 | 35918.01 | 35932.01 | 0 | 0 |  |
| 3 | -17448.6 | 38 | **35156.62**  ****elbow*** | 35047.86 | 35066.86 | **0** | **0** | **0.434** |
| 4 | -17297.8 | 48 | 34923.29 | 34785.91 | 34809.91 | 0.074 | 0.0767 | 0.144 |
| 5 | -17148.9 | 58 | **34693.76** | **34527.75** | **34556.75** | 0.5369 | 0.5412 | 0.142 |
| **COVID Invariance Testing (pre-COVID n=499; COVID n=423)** | | | | | | | | |
| 3-fixed | -18080.3 | 41 | **36440.42** | **36323.06** | **36343.56** | Entropy: 0.934 | | |
| 3-freed | -18058.2 | 68 | 36580.66 | 36385.84 | 36419.84 | Entropy: 0.935 | | |
| **Gender Invariance Testing (n=288 cis-boy, 494 cis-girl, 132 transgender or gender diverse)** | | | | | | | | |
| 3-fixed | -18177.6 | 44 | **36626.70** | 36362.36 | 36366.90 | Entropy: **0.949** | | |
| 3-freed | -18020.49 | 98 | 36709.13 | **36076.63** | **36081.10** | Entropy 0.942 | | |

Figure S2.1

Table S2.2 Independent T-test pre and COVID (defined by March 2020 to present)

|  | Pre-COVID (n=495) | COVID (n=421) | p-value |
| --- | --- | --- | --- |
| Alcohol | 0.78 | 0.88 | 0.155 |
| HED | 0.83 | 0.86 | 0.873 |
| Cannabis | 0.79 | 0.96 | 0.040 |
| Cigarettes/e-cigarettes | 0.82 | 1 | 0.043 |
| Other Drugs | 0.12 | 0.19 | 0.064 |
| SP | 6.63 | 6.75 | 0.505 |
| SAD | 3.74 | 4.39 | 0.006 |
| GAD | 8.00 | 8.39 | 0.059 |
| MDE | 9.95 | 11.08 | <0.001 |
| ADHD | 8.17 | 9.23 | <0.001 |
| ODD | 5.34 | 5.80 | 0.017 |
| CD | 3.12 | 3.32 | 0.368 |

Table S2.3 Class Homogeneity (<0.6 good, >0.9 poor)

|  | **ALC** | **CAN** | **CIG** | **ECIG** | **SP** | **GAD** | **MDE** | **ADHD** | **ODD** | **CD** |
| --- | --- | --- | --- | --- | --- | --- | --- | --- | --- | --- |
| 2LPA | 0.56 | 0.33 | 0.33 | | 1.00 | 0.99 | 0.95 | 0.92 | 0.87 | 0.73 |
| 3LPA | 0.57 | 0.33 | 0.35 | | 0.71 | 0.56 | 0.52 | 0.76 | 0.80 | 0.70 |
| 4LPA | 0.53 | 0.23 | 0.40 | | 0.73 | 0.60 | 0.52 | 0.71 | 0.71 | 0.53 |

Table S2.4 Class Diagnostics (class-invariant diagonal)

|  | n assigned | Posterior class probability (90% CI) | mcaP(k) | AvePP_K_ | OCC_K_ | Entropy |
| --- | --- | --- | --- | --- | --- | --- |
| **2 profile** | | | | | | |
| 1 | 681 | 0.73 (0.71, 0.76) | 0.74 | 0.931 | 4.9 | 0.949 |
| 2 | 241 | 0.27 (0.24, 0.29) | 0.26 | 0.949 | 50.7 |  |
| **3 profile** | | | | | | |
| 1 | 242 | 0.26 (0.24, 0.29) | 0.26 | 0.931 | 37.5 | 0.893 |
| 2 | 444 | 0.48 (0.45, 0.51) | 0.48 | 0.949 | 20.3 |  |
| 3 | 236 | 0.26 (0.23, 0.28) | 0.26 | 0.981 | 149.0 |  |
| **4 profile** | | | | | | |
| 1 | 228 | 0.25 (0.23, 0.27) | 0.25 | 0.931 | 40.5 | 0.887 |
| 2 | 417 | 0.45 (0.42, 0.48) | 0.45 | 0.931 | 16.4 |  |
| 3 | 174 | 0.19 (0.17, 0.21) | 0.19 | 0.931 | 58.8 |  |
| 4 | 103 | 0.11 (0.10, 0.13) | 0.11 | 0.931 | 106.9 |  |

Figures S2.2 Competing Models


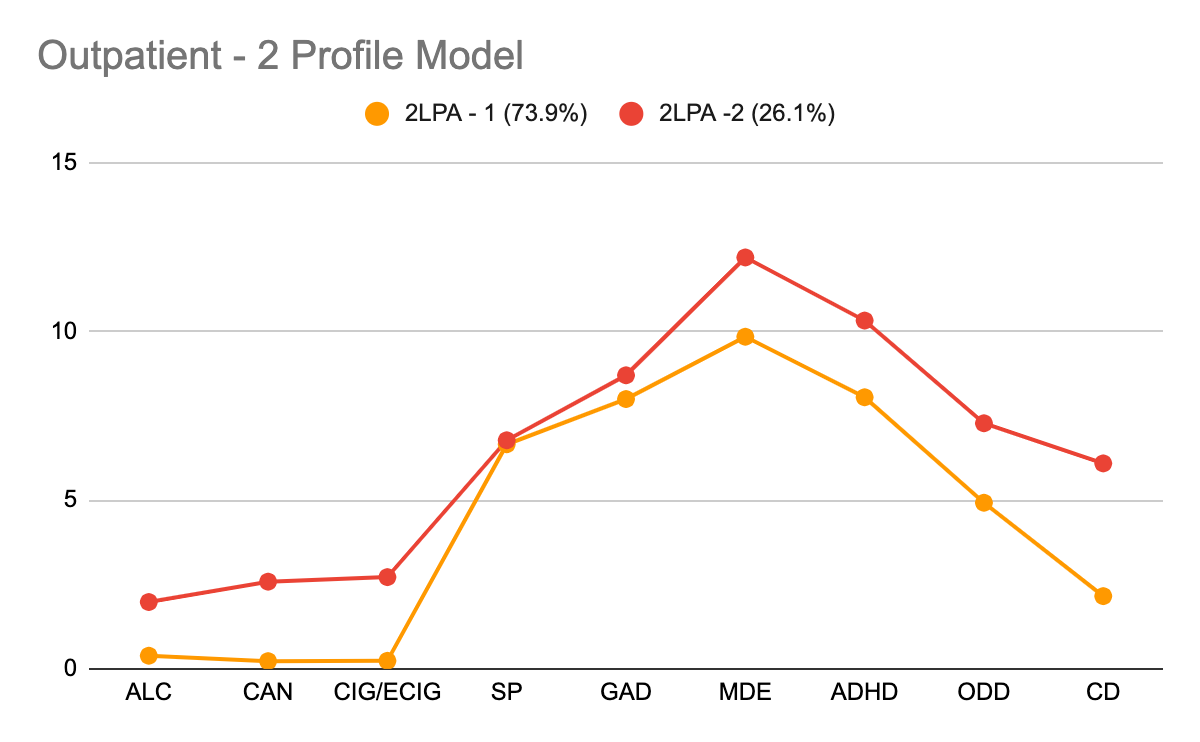


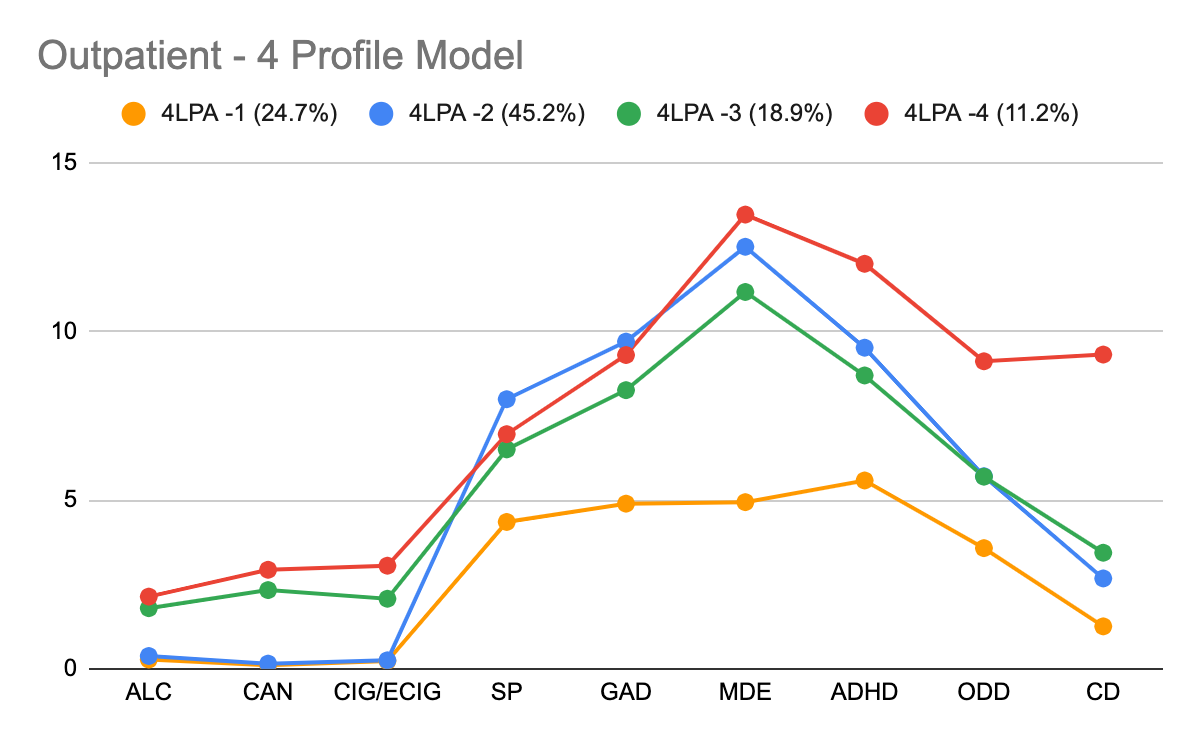


**Figure S2.3 3-Profile Model (raw scores / unstandardized)**

*Note*: The mean (range; 40%/80% percentiles) for the indicators in the full sample for the LPA were: Alcohol 0.8(0-4;0/2), Cannabis 0.9(0-4;0/2), Cig/E-cigarettes 0.9(0-4;0/2), Social Phobia 6.7(0-10;6/9), Generalized Anxiety 8.2(0-12;8/11), Depression 10.5(0-18;10/15), ADHD 8.7(0-16;10/12); Oppositional 5.6(0-12;5/8); Conduct 3.2(0-18;1/5),

Table S2.5 Class separation, comparing standardized mean differences (via cohen’s d) between classes where d<0.85 is a low degree of class separation and **d>2** is high in the final 3-LPA.

| Alcohol Indicator Summary   - Highly separated between low substance use profiles (Low-Low, Low-High) and high substance use profile (High-High) - Low separation between both low substance profiles | | | |
| --- | --- | --- | --- |
|  | Low-High (-SU/+EBS) | High-High (+SU/+EBS) | Low-Low (-SU/-EBS) |
| Low-Low (26.5%) | 0.81 | -0.13 | **-2.07** |
| Low-High(47.8%) | 0.81 | 0.00 | **-1.94** |
| High-High (25.7%) | 0.81 | **1.94** | 0.00 |
| Cannabis Indicator Summary   - Highly separated between low substance use profiles (Low-Low, Low-High) and high substance use profile (High-High) - Low separation between both low substance profiles | | | |
|  | Low-High (-SU/+EBS) | High-High (+SU/+EBS) | Low-Low (-SU/-EBS) |
| Low-Low (26.5%) | 0.73 | -0.05 | **-3.30** |
| Low-High(47.8%) | 0.73 | 0.00 | **-3.24** |
| High-High (25.7%) | 0.73 | **3.24** | 0.00 |
| Cigarette/E-cigarette Indicator Summary   - Highly separated between low substance use profiles (Low-Low, Low-High) and high substance use profile (High-High) - Low separation between both low substance profiles | | | |
|  | Low-High (-SU/+EBS) | High-High (+SU/+EBS) | Low-Low (-SU/-EBS) |
| Low-Low (26.5%) | 0.80 | 0.06 | **-3.06** |
| Low-High(47.8%) | 0.80 | 0.00 | **-3.12** |
| High-High (25.7%) | 0.80 | **3.12** | 0.00 |
| Social Phobia Indicator Summary   - Moderately separate between the lower emotional and behavioural disorder symptom profile (Low-Low) with the higher emotional and behavioural disorder symptom profiles (Low-High, High-High) - Low separation between the higher emotional and behavioural disorder symptom profiles | | | |
|  | Low-High (-SU/+EBS) | High-High (+SU/+EBS) | Low-Low (-SU/-EBS) |
| Low-Low (26.5%) | -1.56 | -1.08 | 0.00 |
| Low-High(47.8%) | 0.00 | 0.49 | 1.56 |
| High-High (25.7%) | -0.49 | 0.00 | 1.08 |
| Generalized Anxiety Disorder Indicator Summary   - High separation between Low-Low and Low-High - Moderate separation between Low-Low and High-High - Low separation between Low-High and High-High | | | |
|  | Low-High (-SU/+EBS) | High-High (+SU/+EBS) | Low-Low (-SU/-EBS) |
| Low-Low (26.5%) | **-2.10** | **-1.71** | 0.00 |
| Low-High(47.8%) | 0.00 | 0.40 | **2.10** |
| High-High (25.7%) | -0.40 | 0.00 | **1.71** |
| Major Depressive Episode Indicator Summary   - Highly separated between the lower emotional and behavioural disorder symptom profile (Low-Low) with the higher emotional and behavioural disorder symptom profiles (Low-High, High-High) - Low separation between the higher emotional and behavioural disorder symptom profiles | | | |
|  | Low-High (-SU/+EBS) | High-High (+SU/+EBS) | Low-Low (-SU/-EBS) |
| Low-Low (26.5%) | **-2.21** | **-2.17** | 0.00 |
| Low-High(47.8%) | 0.00 | 0.04 | **2.21** |
| High-High (25.7%) | -0.04 | 0.00 | **2.17** |
| ADHD Indicator Summary   - Moderately separated between the lower emotional and behavioural disorder symptom profiles(Low-Low) with the higher emotional and behavioural disorder symptom profiles (Low-High, High-High) - Low separation between the higher emotional and behavioural disorder symptom profiles | | | |
|  | Low-High (-SU/+EBS) | High-High (+SU/+EBS) | Low-Low (-SU/-EBS) |
| Low-Low (26.5%) | -1.13 | **-1.46** | 0.00 |
| Low-High(47.8%) | 0.00 | -0.33 | 1.13 |
| High-High (25.7%) | 0.33 | 0.00 | **1.46** |
| ODD Indicator Summary   - Moderately separated between Low-Low and High-High - Low separation between other profiles | | | |
|  | Low-High (-SU/+EBS) | High-High (+SU/+EBS) | Low-Low (-SU/-EBS) |
| Low-Low (26.5%) | -0.71 | **-1.39** | 0.00 |
| Low-High(47.8%) | 0.00 | -0.67 | 0.71 |
| High-High (25.7%) | 0.67 | 0.00 | **1.39** |
| CD Indicator Summary   - Moderately separated between the lower substance use profiles (Low-Low, Low-High) with the higher substance use and emotional and behavioural disorder symptom profiles (High-High) - Low separation between the lower substance use profiles | | | |
|  | Low-High (-SU/+EBS) | High-High (+SU/+EBS) | Low-Low (-SU/-EBS) |
| Low-Low (26.5%) | -0.43 | **-1.71** | 0.00 |
| Low-High(47.8%) | 0.00 | -1.28 | 0.43 |
| High-High (25.7%) | 1.28 | 0.00 | **1.71** |

**Summary of Indicator Separation**: In general, there was high substance indicator separation (as indicated by a standardized mean difference of >=2 reflective of high, and <0.65 reflective of low separation) between profiles in expected directions (i.e., high separation between the +SU/+EBS profile and the other two profiles), moderate to high internalizing and ADHD symptoms in expected directions (i.e., moderate to high separation between the -SU/+EBS and +SU/+EBS profiles with the -SU/-EBS profile), and externalizing indicators only differentiated in the +SU/+EBS profile (low separation between low-low and low-high).

**SM3. Expanded Descriptive and Regression Results**

**Table S3.1. Descriptive statistics** reported as mean(SD) or n(%)

|  | Total (n=927) | -SU/-EBS (n=242) | -SU/+EBS (n=444) | +SU/+EBS (n=236) | Missing |
| --- | --- | --- | --- | --- | --- |
| *Latent Profile Indicators, Mean (standard deviation)* | | | | | |
| Alcohol | 0.8 (1.1) | 0.3 (0.7) | 0.4 (0.8) | 2.0 (1.0) | 11 |
| Cannabis | 0.9 (1.3) | 0.2 (0.6) | 0.3 (0.6) | 2.6 (1.0) | 12 |
| Cigarette/e-cigarette | 0.9 (1.3) | 0.3 (0.7) | 0.3 (0.6) | 2.8 (1.1) | 11 |
| Social Anxiety | 6.7 (2.8) | 4.2 (2.6) | 8.0 (2.1) | 6.8 (2.5) | 9 |
| Generalized Anxiety | 8.2 (3.1) | 4.7 (2.5) | 9.8 (2.0) | 8.8 (2.7) | 9 |
| Depression | 10.5 (4.7) | 5.0 (3.3) | 12.5 (3.2) | 12.3 (3.6) | 9 |
| ADHD | 8.7 (3.7) | 5.7 (3.1) | 9.4 (3.3) | 10.4 (3.0) | 9 |
| Oppositional | 5.6 (2.9) | 3.7 (2.3) | 5.6 (2.7) | 7.4 (2.7) | 9 |
| Conduct | 3.2 (3.3) | 1.4 (1.9) | 2.6 (2.5) | 6.2 (3.9) | 11 |
| *Sociodemographics, Mean (SD) or n(%)* | | | | | |
| Age | 14.7 (1.5) | 14.2 (1.6) | 14.6 (1.4) | 15.5 (1.2) | 0 |
| Cis-gender girl | 495 (53.4%) | 95 (39.3%) | 252 (56.8%) | 147 (62.3%) | 12 |
| Cis-gender boy | 288 (31.1%) | 129 (53.3%) | 92 (20.7%) | 67 (28.4%) | 12 |
| Transgender and Gender Diverse | 132 (14.2%) | 14 (5.8%) | 96 (21.6%) | 22 (9.3%) | 12 |
| LGBTQ status | 393 (42.4%) | 54 (22.3%) | 237 (53.4%) | 101 (42.8%) | 16 |
| Racial Minority | 205 (22.1%) | 44 (18.2%) | 113 (25.5%) | 48 (20.3%) | 99 |
| Income | 5.7 (3.1) | 6.2 (3.3) | 5.5 (2.9) | 5.5 (3.1) | 135 |
| Lifetime other drug use | 71 (7.7%) | 2 (0.8%) | 10 (2.3%) | 59 (25.0%) | 12 |
| *Clinical Risk and Complexity Correlates n(%)* | | | | | |
| Lifetime Abuse | 365 (39.4%) | 44 (18.2%) | 163 (36.7%) | 158 (66.9%) | 10 |
| Lifetime Self Harm | 607 (65.5%) | 77 (31.8%) | 339 (76.4%) | 190 (80.5%) | 13 |
| Lifetime Suicidal Ideation, no attempt | 232 (25.0%) | 38 (15.7%) | 138 (31.1%) | 55 (23.3%) | 17 |
| Lifetime Suicide Attempt | 365 (39.4%) | 40 (16.5%) | 55 (23.3%) | 136 (57.6%) | 17 |
| Lifetime thoughts of hurting or killing others | 224 (24.2%) | 25 (10.3%) | 109 (24.5%) | 90 (38.1%) | 14 |
| Parent Concern: Psychosis | 181 (19.5%) | 36 (14.9%) | 100 (22.5%) | 45 (19.1%) | 79 |
| Past year mental health ED visit | 433 (46.7%) | 75 (31.0%) | 216 (48.6%) | 142 (60.2%) | 7 |

**Table S3.2 Expanded regression results**

|  | +SU/+EBS (ref=-SU/-EBS) | -SU/+EBS (ref==SU/-EBS) | +SU/+EBS (ref=-SU/+EBS) |
| --- | --- | --- | --- |
|  | OR (95% CI); p-values | OR (95% CI); p-values | OR (95% CI); p-values |
| **Sociodemographics** | | | |
| Age | 1.99 (1.71 to 2.32); <0.001 | 1.22 (1.08 to 1.37); 0.001 | 1.63 (1.44 to 1.85); <0.001 |
| Cis-gender girl (ref=cis-boy) | 2.35 (1.52 to 3.64); <0.001 | 2.93 (2 to 4.3); <0.001 | 0.8 (0.54 to 1.2); 0.287 |
| Transgender and Gender Diverse (ref=cis-boy) | 2.25 (0.97 to 5.25); 0.06 | 5.48 (2.67 to 11.26); <0.001 | 0.41 (0.22 to 0.78); 0.006 |
| LGBTQ status | 1.8 (1.12 to 2.89); 0.015 | 2.16 (1.41 to 3.31); <0.001 | 0.83 (0.57 to 1.2); 0.328 |
| Racial Minority | 1.01 (0.6 to 1.7); 0.977 | 1.64 (1.08 to 2.51); 0.021 | 0.61 (0.4 to 0.94); 0.024 |
| Income | 0.92 (0.86 to 0.99); 0.023 | 0.95 (0.9 to 1.01); 0.109 | 0.97 (0.91 to 1.03); 0.299 |
| **Clinical correlates** | | | |
| Youth reported lifetime physical or sexual abuse | 7.49 (4.75 to 11.83); <0.001 | 1.99 (1.32 to 3.01); 0.001 | 3.76 (2.62 to 5.4); <0.001 |
| Youth reported lifetime Self Harm | 6.46 (4.05 to 10.29); <0.001 | 4.48 (3.04 to 6.59); <0.001 | 1.44 (0.93 to 2.23); 0.1 |
| Youth reported lifetime Suicidal Ideation, no attempt (ref=no SI or attempt) | 4.12 (2.33 to 7.31); <0.001 | 4.1 (2.58 to 6.51); <0.001 | 1.01 (0.61 to 1.66); 0.98 |
| Youth reported lifetime Suicide attempt (ref=no SI or attempt) | 8.35 (4.99 to 13.98); <0.001 | 4.19 (2.68 to 6.55); <0.001 | 1.99 (1.26 to 3.17); 0.003 |
| Youth reported lifetime thoughts of hurting or killing others | 7.78 (4.42 to 13.71); <0.001 | 3.03 (1.78 to 5.14); <0.001 | 2.57 (1.75 to 3.77); <0.001 |
| Parent Concern: Psychosis | 1.47 (0.87 to 2.48); 0.149 | 1.48 (0.92 to 2.36); 0.105 | 1 (0.65 to 1.52); 0.985 |
| Youth reported past year MH ED Visit | 2.78 (1.84 to 4.2); <0.001 | 1.73 (1.21 to 2.47); 0.003 | 1.61 (1.14 to 2.26); 0.006 |
